# Influenza A virus shedding reduction observed at 12 weeks post‐vaccination when newborn pigs are administered live‐attenuated influenza virus vaccine

**DOI:** 10.1111/irv.12630

**Published:** 2019-03-06

**Authors:** Troy J. Kaiser, Rex A. Smiley, Brian Fergen, Marc Eichmeyer, Marika Genzow

**Affiliations:** ^1^ Boehringer Ingelheim Vetmedica Inc St. Joseph Missouri; ^2^ Boehringer Ingelheim Vetmedica Inc Ames Iowa; ^3^ Boehringer Ingelheim Vetmedica GmbH Ingelheim Germany

**Keywords:** influenza A, influenza A virus in swine, LAIV, pig, shedding, vaccination

## Abstract

**Background:**

Influenza A virus in swine (IAV‐S) causes an acute respiratory disease of swine which results in great economic losses. A bivalent H1N1 and H3N2, NS1‐truncated live‐attenuated IAV‐S vaccine (LAIV, Ingelvac Provenza^™^) has recently become available.

**Objective:**

Reduction of shedding during an outbreak in the nursery or finisher is an important parameter from an epidemiological control strategy; therefore, a laboratory efficacy study was conducted to evaluate nasal virus shedding when vaccinated pigs were challenged with either heterologous H1N2 or H3N2 strains 12 weeks post‐vaccination.

**Methods:**

Between 1 and 5 days of age, pigs born to IAV‐S seronegative dams were intranasally administered 1 mL of vaccine or saline. At 30 days post‐vaccination, pigs were weaned and randomized into two different challenge groups consisting of vaccinated pigs and control pigs commingled within pens for the two challenge groups. At 85 days post‐vaccination, pigs in the first group were challenged with A/Swine/North Carolina/001169/2006 H1N2 challenge strain, and the second group was challenged with A/Swine/Nebraska/97901‐10/2008 H3N2. Nasal swabs were collected daily for five days and tested by virus isolation.

**Results and conclusion:**

This study showed significant reduction in nasal virus shedding with regard to both frequency and duration. A 1 mL intranasal dose of Ingelvac Provenza^™^ given as early as 1 day of age showed protection for at least 12 weeks later as evidenced by the reduction of shedding live, viable virus after challenge with either a heterologous H1N2 strain or a heterologous H3N2 strain.

## 1 INTRODUCTION

Influenza type A virus (IAV‐S) of the family Orthomyxoviridae causes an acute respiratory disease resulting in distinct economic damages in global pork production. Control strategies include understanding animal movements corresponding to IAV‐S circulation and subsequent establishment of appropriate biosecurity measures including vaccination strategies. Inactivated influenza vaccines are primarily used in adult female breeding swine to provide piglets with antibodies via colostrum which wanes over time not providing protection in the growing pig.^1^ A recent study has shown that piglets play a key role in maintaining IAV‐S in the breeding herds^2^ which provides evidence that early piglet vaccination with a vaccine that provides heterologous protection will contribute to reduce IAV‐S prevalence both within a herd and between herds.

Influenza A virus is mostly transmitted through direct pig‐to‐pig contact and aerosols although other indirect routes of transmission may also exist. Several factors contribute to differences in the transmission dynamics within populations including vaccination, pig flow, animal movement, and animal introduction, among others, which highlights the complexity of influenza A transmission in pigs. In addition, pigs can serve as a reservoir of influenza A viruses for other pigs and other species, and understanding mechanisms of transmission within pigs and from pigs to other species and vice versa is crucial.^3^ Neonatal piglets at birth are immunologically naïve and likely acquire IAV‐S infection while in the farrowing crate.^4^ The role of piglets in maintenance and dissemination of IAV infections has been documented.^4^, ^5^ Vaccination of sows prior to farrowing using inactivated vaccines is the most common protocol in an attempt to mitigate disease of IAV‐S . The results of the study from Diaz et al,^4^ however, contribute to a better understanding of IAV‐S transmission in pigs and indicate the need to focus interventions to control IAV infections on piglets before weaning as well as the young gilt.

Since 2017, a bivalent (H1N1 and H3N2) live‐attenuated influenza virus (LIAV) vaccine in which each fraction is attenuated by truncation of the non‐structural (NS1) protein became commercially available in the United States for use in pigs as young as one day of age (Ingelvac Provenza^™^, Boehringer Ingelheim, St. Joseph, MO). Immunity of this LAIV is not impeded by maternal antibodies in newborn piglets^6^ with regard to frequency and duration of viral shedding. Because pigs can be infected with IAV‐S in both the early nursery and during the finishing periods, the vaccine immunity needed to be investigated with regard to frequency and duration as measured by nasal virus shedding after virulent challenge. To provide evidence‐based epidemiological measures for protection within a population of pigs, the frequency and duration of pigs shedding field virus were investigated.

## 2 MATERIAL AND METHODS

### 2.1 Vaccines and challenge virus

The IAV‐S LAIV was formulated by combining two viruses, the previously described I‐A triple‐reassortant internal gene (TRIG) cluster H3N2^7^ and a novel α‐cluster H1N1 constructed using the same techniques. Once combined, the viruses were lyophilized to create a bivalent vaccine. The vaccine was administered at a 1 mL dose intranasally into one nostril.

IAV‐S challenge isolates γ‐cluster H1N2 (MN MTA, D06‐033775‐4 A/Swine/North Carolina/001169/2006) and cluster IV H3N2 (AB 1‐10, D08‐097901‐10 A/Swine/Nebraska/97901‐10/2008) were propagated in embryonated chicken eggs. At 85 days post‐vaccination, pigs in the first group were administered a 5 mL dose of H1N2 challenge strain at 8.92 log_10_EID_50_/mL, and pigs in the second group were administered a 5 mL dose of the H3N2 challenge strain at 8.16 log_10_EID_50_/mL. The challenge material was kept on ice until administered per the EU Pharmacopeia: Pigs were manually restrained without anesthesia while a catheter was passed just proximal to the tracheal bifurcation for administration of challenge material.

### 2.2 Experimental design

All study procedures and animal care activities were conducted in accordance with the ethical guidelines of the BIAH Institutional Animal Care and Use Committee.

To assess serostatus of the dams, the commercial IAV‐S blocking ELISA (IDEXX Swine Influenza Virus Ab Test; IDEXX Inc., Westbrook, MA) was used by the Iowa State University Veterinary Diagnostic Laboratory (Ames, IA). A sample‐to‐negative control (S/N) ratio less than 0.6 was considered positive, and all 24 dams enrolled into the study had results between 0.785 and 1.284. Additionally, the gilts were screened as negative for IAV‐S H1N1(99 HI) antibodies and for IAV‐S H3N2 (HI c1/3) antibodies by hemagglutination‐inhibition assay (Iowa State University Veterinary Diagnostic Laboratory, Ames, IA). Dams were transported to the Veterinary Resources, Inc. ABL2 isolation facility and randomly assigned to a pen in one of four rooms, two rooms with eight pens each and two rooms with four pens each. Dams farrowed between August 07, 2015, and August 11, 2015. Pigs were processed within 12 hours of birth which included a label dose of Excede for Swine (Zoetis, Lot #4F0622 exp 05/2016) and iron supplementation (Phoenix Pharmaceutical, Lot #150046, exp 01/2019); processing did not include castration, needle‐teeth clipping, or tail docking.

One room of eight litters and one room of four litters were randomized to the vaccine group, and the other two rooms were randomized to the control group. In an effort to ensure all saline‐treated animals remained naive, LAIV and saline vaccinated animals were housed separately. When all pigs were between one and five days of age, all pigs were treated with either vaccine or saline as per the randomization. Pigs in the control group received a 1 mL intranasal dose of saline as control (90 pigs total), and pigs in the vaccine group received a 1 mL intranasal dose of bivalent vaccine rehydrated with diluent (101 pigs total). Pigs were weaned 21 days after vaccination by removal of the sows from crates so that the pigs remained housed with littermates while awaiting randomization for the two challenge phases. Thirty days after vaccination, pigs were randomized to two separate challenge phases: one for H1N2 and one for H3N2. Each separate challenge group consisted of two rooms, one with eight pens and one with four pens.

For the H1N2 challenge, vaccinate and control pigs were commingled by pen, 33 control pigs (representing 11 litters) and 40 vaccinated pigs (representing 11 litters).

For the H3N2 challenge, vaccinated and control pigs were commingled by pen, 37 control pigs (representing 12 litters) and 45 vaccinated pigs (representing 12 litters).

Pigs in both groups were challenged at 85 days post‐vaccination to evaluate on which days post‐challenge (DPC) pigs exposed to heterologous H1N2 or H3N2 virulent challenge in the finishing phase may shed live virus via nasal secretions.

### 2.3 Sampling

Nasal swabs were collected from all pigs on the day prior to challenge and daily for five days post‐challenge (DPC) by insertion of a single swab into each nostril. The swabs were then stored frozen at −70°C in a 5‐mL tube containing 2 mL of tissue culture media formulated with antibiotic and antimycotic until testing could be completed.

### 2.4 Virus isolation

Twenty‐four‐well tissue culture plates of (KEW‐MDCK) cells (a proprietary MDCK cell line stably expressing the Influenza A NS1 gene, which has been useful for detecting low levels of IAV‐S in clinical samples) were seeded with 1 mL of growth media; minimum essential media (MEM) modified (SAFC Cat # 62892‐1000M3056) with 5% fetal bovine serum (SAFC Cat # 12003C‐1000ML) containing 1 × 10^5^ cells. The plates were then incubated for four days at 37°C with 4.5% carbon dioxide. After incubation, the plates were prepared for testing by decanting the growth media and adding back 0.5 mL of wash media (MEM modified without serum but instead containing two units/mL of porcine trypsin [SAFC Cat # T5266‐500 mg]). The plates were then incubated at 37°C with 4.5% carbon dioxide while the samples were being thawed at room temperature. Next, 0.5 to 0.75 mL of each sample was transferred to a centrifuge tube and spun at 10 000 *g* for two minutes in order to pellet sample debris. The wash media was then decanted from the KEW‐MDCK plates, and approximately 100 μL of centrifuged sample was dispensed through a 0.2‐μm filter (Pall Cat # 4602) into each of two duplicate wells. The plates were then incubated at 37°C with 4.5% carbon dioxide for one hour; after this incubation, 0.5 mL of wash media was added back to each well. The plates were then incubated at 37°C with 4.5% carbon dioxide for seven days to allow for low levels of virus to replicate and amplify to levels that can be detected in a hemagglutination assay. After incubation, the supernatant of the duplicate sample wells was harvested and pooled. Then, 50 μL of the pooled material was dispensed into duplicate wells of a 96‐well round bottom plate (BD Falcon Cat # 353910). Washed male turkey red blood cells (Lampire Biological Laboratories Cat # 7209603) diluted to a 0.5% concentration were added to each sample well at 50 μL/well. The plates were sealed and incubated at room temperature for approximately one hour or until the negative control wells formed a button and the positive control wells formed a mat (Table 1).

**Table 1:** Study design

Group		Treatment	Challenge H1N2		Challenge H3N2	
Control	70 pigs	D0 1 mL intranasally	33 pigs	D85 5 mL intratracheally 8.92 log_10_EID_50_/mL	37 pigs	D85 5 mL intratracheally 8.16 log_10_ EID_50_/mL
Vaccine	85 pigs		40 pigs		45 pigs	

### 2.5 Statistics

All data were analyzed with sas 9.4 (SAS Institute, Cary, NC). The results were analyzed by a GLM model that used a binomial distribution with a logit link function, where the number of affected pigs and total number of pigs from each litter within pen were incorporated as the unit of analysis. The null hypothesis was that shedding of virus in nasal swabs in the vaccinated group was not different to the control group. The level of significance was set *P* ≤ 0.05.

## 3 RESULTS

### 3.1 H1N2 challenge: virus isolation of nasal swabs

**Figure 1:**
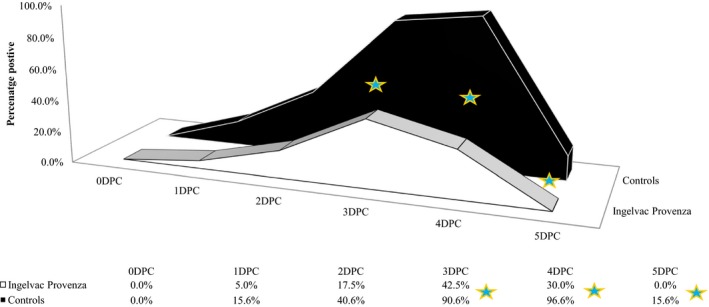
H1N2 Challenge: Percent of pigs with virus isolation‐positive nasal swabs. DPC, days post‐challenge; ★ *P*‐value ≤ 0.014 (Comparison of group proportions)

All pigs were VI negative for nasal swabs prior to challenge, and significantly more control pigs were shedding virus than vaccinated pigs at 3DPC, 4DPC, and 5DPC (*P *≤* *0.014; Figure 1). All control pigs but one were VI positive on at least one nasal swab (96.9% positive) during the five‐day challenge period, while only 53% of vaccinated pigs were ever positive, a significant difference (*P *=* *0.011, Table 2). In the vaccinated group, virus shedding peaked 3DPC with 42.5% of pigs positive and ceased after 4DPC. The control group peaked 4DPC with 96.9% positive and still had 15.6% pigs VI positive on nasal swabs at the end of the challenge phase, 5DPC. The mean duration of shedding was significantly reduced from 2.59 days in control pigs to 0.95 days in vaccinated pigs (*P *<* *0.0001).

**Table 2:** Proportion of pigs ever virus isolation (VI) positive for nasal swab after H1N2 challenge 12 weeks post‐vaccination

Treatment	Ever positive for nasal swab VI	Positive on last day	Proportion positive (95 CL)	*P*‐value
Control	31/32 (97%)	5/32 (16%)	0.97 (0.73, 1.00)	0.011
Vaccine	21/40 (53%)	0/40 (0%)	0.53 (0.34, 0.70)	

95CL, upper and lower 95% confidence limits; VI, virus isolation.

### 3.2 H3N2 challenge: Virus isolation of nasal swabs

**Figure 2:**
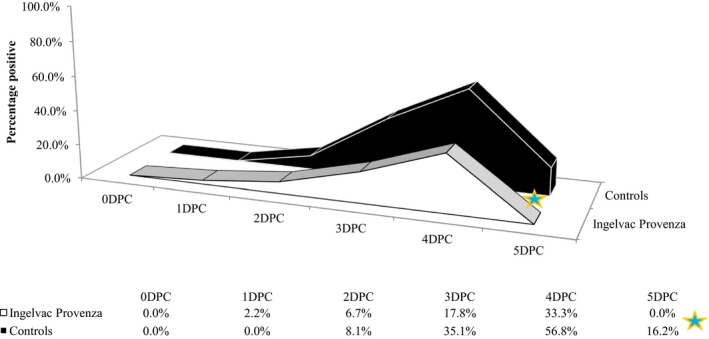
H3N2 Challenge: Percent of pigs with virus isolation‐positive nasal swabs. DPC, days post‐challenge; ★ *P*‐value = 0.007(Comparison of group proportions)

All pigs were VI negative for nasal swabs prior to challenge, and significantly more control pigs were shedding virus than vaccinated pigs at 5DPC (*P *≤* *0.007; Figure 2). Over the five‐day challenge period, 42% of vaccinated pigs were VI positive from at least one nasal swab, compared to 65% of control pigs, a significant difference (*P *=* *0.029, Table 3). The proportion of VI positive pigs in the vaccinated group peaked at 4DPC with 33.3%. By 5DPC, the vaccinated group had quit shedding, while the control group still had 16.2% pigs positive. The mean duration of shedding was significantly reduced from 1.16 days for control pigs to 0.62 days for vaccinated pigs (*P *=* *0.0484).

**Table 3:** Proportion of pigs ever virus isolation (VI) positive for nasal swab after H3N2 challenge 12 weeks post‐vaccination

Treatment	Ever positive for nasal swab VI	Positive on last day	Proportion positive (95CL)	*P*‐value
Control	24/37 (65%)	6/37 (16%)	0.65 (0.50, 0.78)	0.029
Vaccine	19/45 (42%)	0/45 (0%)	0.42 (0.30, 0.56)	

95CL, upper and lower 95% confidence limits; VI, virus isolation.

## 4 DISCUSSION

Recent modeling and empirical work on IAV‐S suggests piglets play an important role as an endemic reservoir.^2^ The current standard of vaccination is using whole killed virus vaccines (KV), whereby KVs are only effective if the vaccine strains closely match the currently circulating strains in pigs. KVs are used primarily in breeding females to confer passive immunity to their offspring which wanes over time. Additionally, this passive immunity does not protect piglets against infection and transmission of IAV‐S but rather against clinical disease.^8^ Recently, a LAIV vaccine (Ingelvac Provenza^™^) has been reported to be a valuable tool in reducing viral shedding in heterologous challenges in young piglets both with and without maternal antibodies.^6^, ^9^ Intranasal LAIV vaccines given to newborn piglets have the ability to induce a broad, cross‐protective immune response similar to natural infection.^9^ The protective local immunity without the risk of VAERD (vaccine‐associated enhanced respiratory disease) may include priming of cell‐mediated immunity.^10^ The objective of this study was to investigate whether vaccination with this LAIV would reduce viral shedding with regard to both frequency and duration in a population of finishing pigs. A 1 mL intranasal dose of Ingelvac Provenza^™^ given as early as 1 day of age showed protection for at least 12 weeks later as evidenced by the reduction of shedding live, viable virus after challenge with either a heterologous H1N2 strain or a heterologous H3N2 strain. The 12‐week time frame is important as it corresponds with risk for IAV exposure in the early finishing phase of production. This study demonstrated efficacy as measured by reduction in viral shedding in pigs directly vaccinated showing an affect that lasts into the finishing phase, rather than relying on passive immunity or waiting to vaccinate until maternal antibody has waned. These findings are in contrast to study results when the breeding herd is vaccinated with KVs, where vaccination alone clearly does not significantly reduce the level of infectious piglets^2^ and suggest that piglets should be targeted for further intervention to prevent the long‐term persistence of influenza infection within a farm. With the findings of our study, further research is warranted whether intranasal vaccination with this LAIV (Ingelvac Provenza^™^) can significantly reduce shedding in endemically infected herds.
